# Lemierre's Syndrome Following Streptococcus anginosus Pharyngitis: An Unusual Case of a Forgotten Disease

**DOI:** 10.7759/cureus.80658

**Published:** 2025-03-16

**Authors:** Debarghya Chakraborty, Humayra Tabassum, Sowmitra Das

**Affiliations:** 1 Internal Medicine, King's College University Hospital Trust, London, GBR

**Keywords:** acute pharyngitis, atypical lemierre's syndrome, internal jugular vein thrombophlebitis, streptococcus anginosus group, thrombus dissemination

## Abstract

Lemierre's syndrome is a rare complication of oropharyngeal infection characterized by septic thrombophlebitis in the internal jugular vein. Despite this being a common occurrence before the era of antibiotics, with beta-lactam antibiotic usage, this pathology has been a rare occurrence since the mid-1900s. Most cases are attributed to *Fusobacterium* bacteraemia. However, in this case report, we present a middle-aged patient diagnosed with Lemierre's syndrome secondary to an atypical pharyngeal infection of *Streptococcus anginosus.* A computed tomography (CT) scan of the neck visualized the internal jugular vein thrombus, aiding in the diagnosis of the condition. He further develops pulmonary complications from the dissemination of thrombus from the internal jugular vein. This case emphasizes the need for higher clinical suspicion to ensure timely management of the condition. Similarly, it demonstrated how a multidisciplinary approach and appropriate follow-up are crucial in the successful management of these patients. It also highlights the requirement for larger population-based studies to ensure definitive and evidence-based clinical guidelines to reduce the morbidity and mortality from this rare yet serious condition of Lemierre's syndrome.

## Introduction

Lemierre's syndrome is a rare complication of oropharyngeal infection characterized by septic thrombophlebitis in the internal jugular vein (IJV). The incidence of this rare disease is only 3.6 cases per one million cases per year [1]. Due to the condition's rarity, only a few risk factors have been identified. Studies have indicated a higher prevalence of young males developing this condition [2]. Immunosuppression and poor oral hygiene have also been shown to contribute as risk factors to Lemierre's syndrome [3,4].

Around one-fourth of these cases are attributed to the anaerobic gram-negative bacillus *Fusobacterium*, the most commonly occurring species being *Fusobacterium necrophorum* [5]. Despite this being a common occurrence before the era of antibiotics, with beta-lactam drug usage, this pathology has been a rare occurrence since the mid-1900s, especially in immunocompetent patients [6]. But recently, a surge in the incidence of Lemierre's syndrome is worrying the medical fraternity. One of the suggested reasons behind this is the lack of empirical use of antibiotics in the oropharyngeal infection [6].

Progression of the acute infection further from the oropharynx leads to extensive inflammation in the parapharyngeal space and surrounding soft tissue. Extravascular compression from soft tissue oedema combined with spreading intravascular infection leads to venous stasis and consequently forms a thrombus at the peri-tonsillar vein and IJV [7]. This gets further complicated as a septic embolus can disseminate to other organs, most commonly the lungs, resulting in end-organ damage.

Septic thrombophlebitis in patients with Lemierre's syndrome presents as unilateral neck swelling and tenderness, extending up to the mandibular angle, along with fever post-one week of initial illness onset [8]. Blood results reveal high infection markers with a potential positive anaerobic blood culture. The presence of a thrombus in the IJV is visualized by computed tomography (CT), aiding in diagnosis [5]. This is followed by a CT scan of the lung fields to detect any further complications like septic emboli or abscesses secondary to primary thrombus dissemination. A combination of antibiotics is principally used for treatment [8]. For refractory or progressive disease, anticoagulants are also considered. This case report describes an atypical presentation of Lemierre's syndrome caused by *Streptococcus anginosus *rather than the usual *Fusobacterium* species, demonstrating the need for broader diagnostic suspicion. It also highlights how the involvement of a multidisciplinary team ensures successful treatment and follow-up of this rare but serious condition.

## Case presentation

A 42-year-old male patient presented to the emergency department with complaints of fever and sore throat for over one week without any therapeutic relief with conservative management. He also reported a gradual development of difficulty swallowing. He denied any associated cough, chest pain, or shortness of breath. His social history was unremarkable, with no unwell contact or recent travel. He had no significant or relevant past medical history.

The initial observation revealed a body temperature of 39.8°C with tachycardia of 102 beats per minute. The initial examination revealed redness at the back of the throat without the presence of enlarged tonsils, peritonsillar abscess, or quinsy. Normal vesicular breathing sounds without any added sounds were found. Raised infection markers, i.e., neutrophilic leukocytosis, raised procalcitonin, and elevated C-reactive protein levels, were found in blood parameters (Table 1). Marked neutrophilia and thrombocytopenia indicated a possible septicemia. Blood cultures were sent for analysis. The impression of the overall clinical picture at this stage was pharyngitis. The patient was started on benzylpenicillin 1.2 g four times daily.

**Table 1:** Laboratory results at the time of admission Marked neutrophilia and thrombocytopenia, along with high procalcitonin and C-reactive protein levels, indicate severe infection and possible septicemia.

Parameter	Results	Normal Reference Range
Haemoglobin	169 g/L	133-167 g/L
White blood cells	26.7 × 10^9^/L	2.9-9.6 × 10^9^/L
Neutrophils	124.5 × 10^9^/L	1.50-6.10 × 10^9^/L
Lymphocytes	0.98 × 10^9^/L	0.80-3.50 × 10^9^/L
Platelets	71 × 10^9^/L	140-400 ×10^9^/L
Prothrombin time	12.3 seconds	10.1-11.9 seconds
Activated partial thromboplastin time	26.1 seconds	21.1-28.5 seconds
International normalized ratio (INR)	1.2	0.8-1.2
Sodium	131 mmol/L	135-145 mmol/L
Potassium	3.4 mmol/L	3.5-5.3 mmol/L
Urea	4.3 mg/dL	16-48 mg/dL
Creatinine	49 µmol/L	61-123 µmol/L
Alanine aminotransferase	18 U/L	10-35 U/L
Total bilirubin	23 µmol/L	<21 µmol/L
C-reactive protein	139 mg/L	<5 mg/L
Procalcitonin	3.14 µg/L	<0.06 µg/L

Blood cultures from three days later showed growth of Gram-positive cocci in chains (*Streptococcus anginosus*). During this period, the patient kept on having regular temperature spikes. Based on culture sensitivity and ongoing pyrexia, he was switched to vancomycin. The impression after discussion with the microbiology consultant was that the patient might have a deep-seated infection or intra-abdominal collection. However, a negative CT of the abdomen and pelvis ruled out any intra-abdominal collection. The absence of peripheral stigmata of infective endocarditis or any cardiac murmurs and a negative bedside transthoracic echocardiogram showing no images suggestive of vegetation ruled out infective endocarditis. The clinical examination of the back of the throat showed the absence of uvula deviation and swelling in the peritonsillar area and soft palate, which ruled out peritonsillar abscess or quinsy.

Two days later, the patient developed swelling at the left anterior neck with mild tenderness. The examination revealed matted left anterior cervical lymph nodes, mildly tender, firm in consistency, mobile, and not attached to underlying structures, the biggest measuring approximately 3x4 cm. With worsening symptoms and still ongoing pyrexia of unknown origin, he was switched to IV clindamycin 600 mg and benzylpenicillin 2.4 g, both four times daily. An ultrasound scan (USS) of the neck and a CT scan of the neck and lungs were ordered to rule out any abscess collection, infected lymph nodes, or thrombophlebitis.

**Figure 1:**
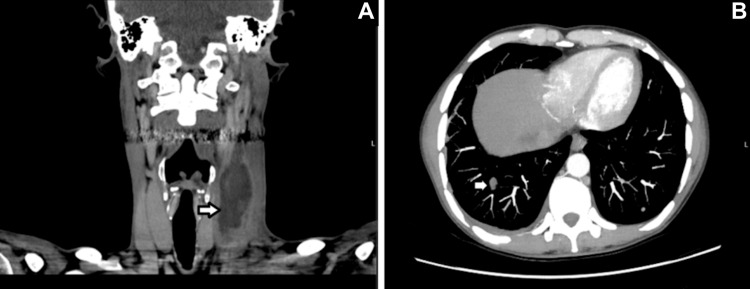
CT neck and thorax before anticoagulation CT neck (A) shows a thrombus within the left internal jugular vein measuring 2.6 x 2.6 x 8.2 cm (white arrow). CT thorax (B) shows a right basal cavitating lesion, which is concerning for septic embolization (white arrow).

Neck USS showed an enlarged structure in the left lower neck along the course of the jugular vein, and thrombosis of the jugular vein was suspected. A CT scan of the neck (Figure 1A) and thorax (Figure 1B) was done immediately, showing a septic mass in the left IJV and multiple septic emboli in both lungs. Lemierre's syndrome, as a complication from pharyngitis, was diagnosed at this point.

**Figure 2:**
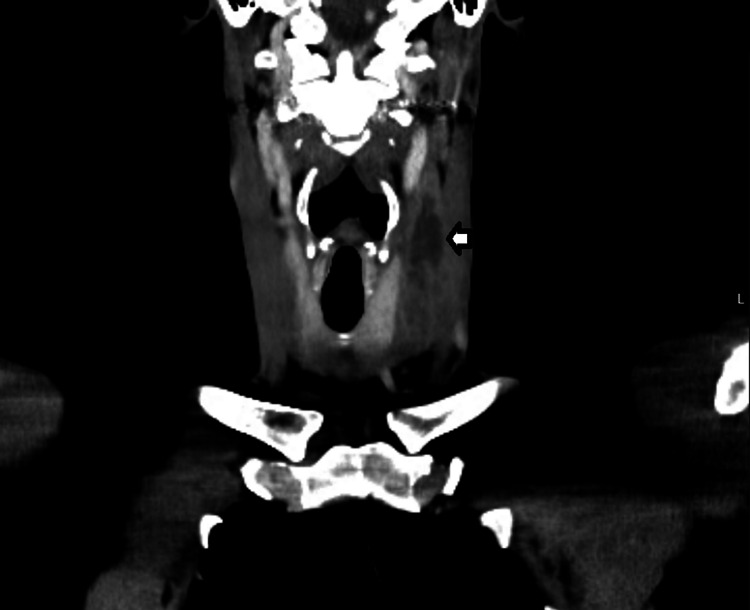
CT neck one week post-anticoagulation The CT neck shows a thrombus within the left internal jugular vein, which appears smaller in size, measuring 2 x 2 x 6.7 cm (white arrow).

After consultation with a multidisciplinary team comprising senior clinicians from microbiology, vascular surgery, and haematology, he was started on enoxaparin 1 mg/kg/day along with aspirin 75 mg once daily and continued with the same previously prescribed antibiotics. After one week, a repeat CT scan of the neck (Figure 2) was done to see the progression of the previous abnormal findings. Regression in the size of the left IJV thrombus was noted.

He was discharged from the hospital two days later with aspirin 75 mg once daily for six weeks, oral co-amoxiclav 625 mg (amoxicillin 500 mg and clavulanic acid 125 mg) thrice daily for two weeks, apixaban 5 mg tablet twice daily, and a follow-up planned with the haematology team in one month. During the follow-up clinic visit, the patient reported that previous symptoms had resolved without any new complications. Further follow-up in the clinic was arranged in three months.

## Discussion

Here, we report a case of Lemierre's syndrome due to an atypical microorganism diagnosed late in the course of medical management. In most cases, one of the major constraints of diagnosing this rare but serious complication is low clinical suspicion. To avoid any delay in diagnosis, high clinical suspicion based on presented signs and symptoms, especially in the presence of any risk factors, is crucial.

Lemierre's syndrome usually presents after one week of worsening oropharyngeal infection. A degree of immunosuppression due to poorly controlled diabetes or oropharyngeal malignancy and increased use of non-steroidal anti-inflammatory drugs contributing to poor oral health can increase the risk of developing this complication [3,4].

The majority of Lemierre's syndrome cases are yet recorded as a result of *Fusobacterium* bacteraemia. Only in a minority of cases are organisms such as *E. coli*, *Staphylococcus*, and *Streptococcus* found as causative organisms [9]. Here, we report an atypical case of Lemierre's syndrome following pharyngitis with *Streptococcus anginosus* bacteraemia.

Similar to this case report, a small number of recent studies have also reported Lemierre's syndrome incidence secondary to *Streptococcus anginosus* infection [10]. A recent study focusing on infectious disease complications recorded increasing rates of intracranial complications secondary to sinusitis and pharyngitis from *Streptococcus anginosus* bacteraemia [11]. Bigger population studies are required to investigate whether Lemierre's syndrome incidence follows a similar trend of increased *Streptococcus anginosus* infection. Additionally, the absence of *Fusobacterium *growth can be due to either culture samples taken after the commencement of antibiotics or, generally, it being difficult to culture as it requires a strict anaerobic environment [12]. An interesting report suggested that the coexistence of *Streptococcus anginosus* with classic *Fusobacterium* aids in accelerated inflammation, as evidenced in Lemierre's syndrome [13]. Thus, isolating *Streptococcus anginosus* in blood culture should increase clinical suspicion of Lemierre's syndrome.

The presentation of unilateral tender neck mass between two heads of the sternocleidomastoid muscle on the background of a prolonged oropharyngeal infection indicates not just Lemierre's syndrome but also a wide set of differentials, such as cervical lymphadenitis and neck abscess. Imaging helps to rule out other differentials and confirm the clinical suspicion. In our case, the clinical suspicion based on new-onset neck pain was confirmed by a CT scan of the neck and led to the definitive diagnosis of Lemierre's syndrome. Although ultrasound is an easy, accessible, and cost-effective method, it has less sensitivity in diagnosing IJV thrombosis, making it a less optimum imaging modality. CT scan has emerged as the most used diagnostic imaging modality for Lemierre's syndrome, compared to high-cost MRI, even though both can demonstrate pathology.

After appropriate diagnosis by imaging, evidence-based treatment is crucial. Broad-spectrum antibiotics with anaerobic coverage are the definitive treatment for Lemierre's syndrome. However, due to the rarity of the condition, the appropriateness of using anticoagulation is not clear according to clinical guidance. In this patient, enoxaparin (low molecular weight heparin) was used along with antiplatelet aspirin, and a gradual reduction of the thrombus was achieved. A population-based study involving 82 patients conducted in 2020 showed that no adverse and therapeutic outcome differences were recorded between patients with or without anticoagulation use [14]. However, a recent case report has indicated that anticoagulation therapy might increase the efficacy of antimicrobial therapy used in Lemierre's syndrome [15]. Whether the use of anticoagulant agents dissolves the clot and reduces local oedema and consequently ensures better penetration of antibiotics to the bacteria trapped in the thrombus is not clear [16].

Keeping in mind the high mortality rate of 4%, a European collaborative study comprising 712 patients concluded that in case of no major contraindication like active bleeding, anticoagulation should be added with antibacterial treatment [17]. Similar larger population studies are required to ensure evidence-based guidelines for the treatment of Lemierre's syndrome are established. This study also highlighted the requirement of involving a multidisciplinary team comprising microbiology and haematology physicians in immediate management. In case of an abscess found in imaging, the surgical team might be required to be involved in incision and drainage [17]. This disease also records as high as 12% of cases ending up with long-term respiratory and neurological complications [17]. Thus, appropriate and timely follow-up is crucial for this patient group. In the present case, with a long course of antibiotics and anticoagulation and finally follow-up from multiple specialities, clinical recovery was achieved.

## Conclusions

Lemierre's syndrome is a rare yet serious medical presentation carrying high mortality and morbidity risk. This report presents a case of a middle-aged patient diagnosed with Lemierre's syndrome secondary to an atypical Streptococcus anginosus infection. He further developed pulmonary complications secondary to thrombus formation in the IJV. This case emphasizes the need for higher clinical suspicion to ensure timely management of the condition. Similarly, it demonstrated how a multidisciplinary approach and appropriate follow-up are crucial in the successful management of these patients. It also highlights the requirement for larger population-based studies to ensure definitive and evidence-based clinical guidelines to reduce the morbidity and mortality from this rare yet serious condition of Lemierre's syndrome.
